# 2-[(1R*,4R*)-1,4-Dihy­droxy­cyclo­hex­yl]acetic acid

**DOI:** 10.1107/S1600536811010397

**Published:** 2011-03-26

**Authors:** Mohammad Arfan, Syed Hamid Hussain, M. Nawaz Tahir, Jamal Rafique, Farzana Shaheen

**Affiliations:** a Institute of Chemical Sciences, University of Peshawar, Peshawar, Pakistan; b Department of Physics, University of Sargodha, Sargodha, Pakistan; c International Centre for Chemical and Biological Sciences, HEJ Research Institute of Chemistry, University of Karachi, Pakistan

## Abstract

The title compound, C_8_H_14_O_4_, is an isolation product of the aerial parts of *Senecio desfontanei*. The acetic acid group is oriented at a dihedral angle of 48.03 (9)° with respect to the basal plane of the cyclo­hexane-1,4-diol chair. An intra­molecular O—H⋯O hydrogen bond generates an *S*(6) ring with an envelope conformation. In the crystal, mol­ecules are linked by O—H⋯O hydrogen bonds, resulting in *R*_3_^3^(20) ring motifs and *C*(2) O—H⋯O—H⋯O—H⋯ chains. Overall, a three-dimensional polymeric network arises. A C—H⋯O contact is also present.

## Related literature

For related structures, see: Jasinski *et al.* (2009 ▶); Vasudev *et al.* (2008 ▶). For graph-set notation, see: Bernstein *et al.* (1995 ▶).
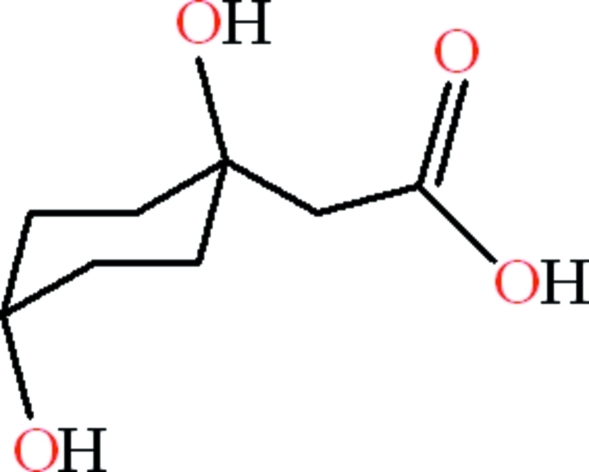


## Experimental

### 

#### Crystal data

C_8_H_14_O_4_
*M* *_r_* = 174.19
Triclinic, 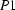

*a* = 5.7301 (4) Å
*b* = 6.3493 (3) Å
*c* = 6.4964 (4) Å
α = 92.863 (2)°
β = 97.223 (1)°
γ = 108.258 (2)°
*V* = 221.67 (2) Å^3^
*Z* = 1
Mo *K*α radiation
μ = 0.10 mm^−1^
*T* = 296 K
0.28 × 0.12 × 0.10 mm

#### Data collection

Bruker Kappa APEXII CCD diffractometer
Absorption correction: multi-scan (*SADABS*; Bruker, 2005 ▶) *T*_min_ = 0.935, *T*_max_ = 0.965
3664 measured reflections
1092 independent reflections
932 reflections with *I* > 2σ(*I*)
*R*_int_ = 0.025

#### Refinement

*R*[*F* ^2^ > 2σ(*F* ^2^)] = 0.037
*wR*(*F* ^2^) = 0.091
*S* = 1.07
1092 reflections
118 parameters
H atoms treated by a mixture of independent and constrained refinement
Δρ_max_ = 0.22 e Å^−3^
Δρ_min_ = −0.15 e Å^−3^

### 

Data collection: *APEX2* (Bruker, 2009 ▶); cell refinement: *SAINT* (Bruker, 2009 ▶); data reduction: *SAINT*; program(s) used to solve structure: *SHELXS97* (Sheldrick, 2008 ▶); program(s) used to refine structure: *SHELXL97* (Sheldrick, 2008 ▶); molecular graphics: *ORTEP-3 for Windows* (Farrugia, 1997 ▶) and *PLATON* (Spek, 2009 ▶); software used to prepare material for publication: *WinGX* (Farrugia, 1999 ▶) and *PLATON*.

## Supplementary Material

Crystal structure: contains datablocks global, I. DOI: 10.1107/S1600536811010397/hb5819sup1.cif

Structure factors: contains datablocks I. DOI: 10.1107/S1600536811010397/hb5819Isup2.hkl

Additional supplementary materials:  crystallographic information; 3D view; checkCIF report

## Figures and Tables

**Table 1:** Hydrogen-bond geometry (Å, °)

*D*—H⋯*A*	*D*—H	H⋯*A*	*D*⋯*A*	*D*—H⋯*A*
O1—H1⋯O3^i^	0.83 (3)	1.78 (3)	2.608 (3)	177 (3)
O3—H3⋯O4^ii^	0.86 (3)	1.90 (3)	2.756 (2)	175 (3)
O4—H4⋯O1^iii^	0.83 (3)	2.39 (3)	3.007 (3)	131 (3)
O4—H4⋯O2	0.83 (3)	2.20 (3)	2.789 (3)	128 (3)
C5—H5*A*⋯O1^iv^	0.97	2.60	3.511 (3)	157

Symmetry codes: (i) 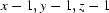; (ii) 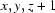; (iii) 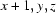; (iv) 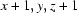.

**Fig. 1.**
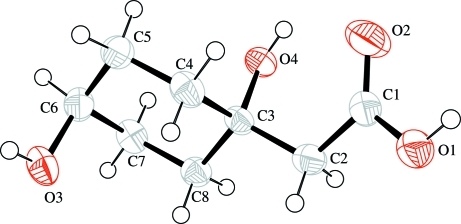
View of the title compound with displacement ellipsoids drawn at the 50% probability level.

**Fig. 2.**
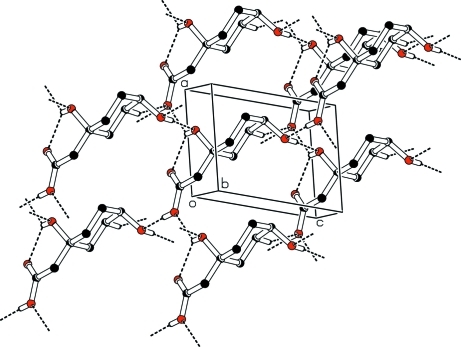
The partial packing of the title compound, which shows that molecules form polymeric network with ring motifs.

## supplementary crystallographic information

### Comment

The crystal structure of (1-(aminomethyl)cyclohexane)acetic acid hydrochloride hemihydrate (Jasinski *et al.*, 2009) and 1-ammoniocyclohexaneacetic acid chloride monohydrate (Vasudev *et al.*, 2008) have been published which are related to the title compound (I, Fig. 1).

In (I), there are cyclohexane-1,4-diol and acetic acid moieties. The basal plane of cyclohexane A (C4,C5,C7,C8) and the acetic acid moiety B (O1/C1/C2/O2) are planar with r. m. s. deviation of 0.0022 and 0.0016 Å, respectively. The dihedral angle between A/B is 48.03 (9)°. The hydroxy atoms O3 and O4 are at a distance of 2.0320 (26) and -2.0535 (23) Å respectively, from the basal plane whereas the C-atoms C3 and C6 are at a distance of -0.6862 (29) and -0.6195 (23) Å respectively, from it. There exist an intra molecular H-bonding of O—H···O type (Table 1, Fig. 2) forming an S(6) and *R*_3_^3^(20) ring motifs (Bernstein *et al.*, 1995). The molecules are stabilized in the form of three dimensional polymeric network with O—H···O—H···O—H··· chains (Fig. 2).

### Experimental

The air dried and pulverized aerial parts of Senecio desfontanei (12 kg), collected from Kaghan, KPK, Pakistan, in July 2008, were subjected to cold extraction with methanol (MeOH) in percolator. The MeOH extract was concentrated *in vacuo* to give dark greenish crude extract (300 g) which was then suspended in distilled water and successively partitioned with n-hexane, dichloromethane (DCM), ethyl acetate (EtOAc). The EtOAc fraction (90 g) was subjected to column chromatography (CC) on silica gel and n-hexane: EtOAc (100:0 → 0:100) as eluting system. This resulted in total of 20 subfractions *i.e.* 1 A-20 A compiled on the basis of TLC profiles. Subfraction 12 A was resubjected to CC on silica gel and eluted with n-hexane: EtOAc (20:80) yielding a crystalline compound containing minor impurities. The impurity was washed off with DCM. Transparent needles of the title compound were obtained by recrystallization using a mixture of EtOAc:MeOH (85:15).

### Refinement

In the absence of significant anomolous scattering, the Friedal pairs were merged before refinement.

The coordinates of the hydroxy H-atoms were refined. The H-atoms were positioned geometrically (O—H = 0.82, C–H = 0.97–0.98 Å) and refined as riding with *U*_iso_(H) = x*U*_eq_(C, O), where x = 1.2 for all H-atoms.

### Crystal data

**Table:**

C_8_H_14_O_4_	*Z* = 1
*M**_r_* = 174.19	*F*(000) = 94
Triclinic, *P*1	*D*_x_ = 1.305 Mg m^−^^3^
Hall symbol: P 1	Mo *K*α radiation, λ = 0.71073 Å
*a* = 5.7301 (4) Å	Cell parameters from 933 reflections
*b* = 6.3493 (3) Å	θ = 3.2–28.4°
*c* = 6.4964 (4) Å	µ = 0.10 mm^−^^1^
α = 92.863 (2)°	*T* = 296 K
β = 97.223 (1)°	Needle, colorless
γ = 108.258 (2)°	0.28 × 0.12 × 0.10 mm
*V* = 221.67 (2) Å^3^	

### Data collection

**Table:**

Bruker Kappa APEXII CCD diffractometer	1092 independent reflections
Radiation source: fine-focus sealed tube	932 reflections with *I* > 2σ(*I*)
graphite	*R*_int_ = 0.025
Detector resolution: 7.50 pixels mm^-1^	θ_max_ = 28.4°, θ_min_ = 3.2°
ω scans	*h* = −7→7
Absorption correction: multi-scan (*SADABS*; Bruker, 2005)	*k* = −8→8
*T*_min_ = 0.935, *T*_max_ = 0.965	*l* = −8→8
3664 measured reflections	

### Refinement

**Table:**

Refinement on *F*^2^	Primary atom site location: structure-invariant direct methods
Least-squares matrix: full	Secondary atom site location: difference Fourier map
*R*[*F*^2^ > 2σ(*F*^2^)] = 0.037	Hydrogen site location: inferred from neighbouring sites
*wR*(*F*^2^) = 0.091	H atoms treated by a mixture of independent and constrained refinement
*S* = 1.07	*w* = 1/[σ^2^(*F*_o_^2^) + (0.0539*P*)^2^ + 0.0008*P*] where *P* = (*F*_o_^2^ + 2*F*_c_^2^)/3
1092 reflections	(Δ/σ)_max_ < 0.001
118 parameters	Δρ_max_ = 0.22 e Å^−^^3^
0 restraints	Δρ_min_ = −0.15 e Å^−^^3^

### Special details

**Table:**

Geometry. Bond distances, angles *etc*. have been calculated using the rounded fractional coordinates. All su's are estimated from the variances of the (full) variance-covariance matrix. The cell e.s.d.'s are taken into account in the estimation of distances, angles and torsion angles
Refinement. Refinement of *F*^2^ against ALL reflections. The weighted *R*-factor *wR* and goodness of fit *S* are based on *F*^2^, conventional *R*-factors *R* are based on *F*, with *F* set to zero for negative *F*^2^. The threshold expression of *F*^2^ > σ(*F*^2^) is used only for calculating *R*-factors(gt) *etc*. and is not relevant to the choice of reflections for refinement. *R*-factors based on *F*^2^ are statistically about twice as large as those based on *F*, and *R*- factors based on ALL data will be even larger.

### Fractional atomic coordinates and isotropic or equivalent isotropic displacement parameters (Å^2^)

**Table:**

	*x*	*y*	*z*	*U*_iso_*/*U*_eq_	
O1	−0.1847 (3)	0.0914 (3)	−0.1705 (3)	0.0440 (5)	
O2	0.1853 (4)	0.0521 (4)	−0.1685 (4)	0.0773 (9)	
O3	0.6317 (3)	0.6910 (3)	0.6378 (3)	0.0431 (5)	
O4	0.5902 (3)	0.4332 (3)	−0.0313 (2)	0.0359 (5)	
C1	0.0589 (4)	0.1643 (4)	−0.1287 (3)	0.0417 (7)	
C2	0.1589 (4)	0.4007 (4)	−0.0290 (4)	0.0401 (7)	
C3	0.4158 (4)	0.4631 (3)	0.1003 (3)	0.0304 (6)	
C4	0.4181 (4)	0.3231 (3)	0.2851 (3)	0.0382 (7)	
C5	0.6749 (5)	0.3888 (4)	0.4164 (4)	0.0414 (7)	
C6	0.7759 (4)	0.6356 (4)	0.4899 (3)	0.0391 (7)	
C7	0.7640 (4)	0.7782 (4)	0.3096 (4)	0.0379 (7)	
C8	0.5067 (4)	0.7087 (3)	0.1812 (3)	0.0341 (7)	
H1	−0.238 (5)	−0.036 (5)	−0.231 (5)	0.0528*	
H2A	0.04354	0.42391	0.05989	0.0481*	
H2B	0.16539	0.50069	−0.13778	0.0481*	
H3	0.627 (5)	0.610 (5)	0.740 (5)	0.0517*	
H4	0.556 (5)	0.301 (5)	−0.077 (4)	0.0430*	
H4A	0.29895	0.34223	0.37184	0.0458*	
H4B	0.36752	0.16699	0.23368	0.0458*	
H5A	0.66520	0.30369	0.53680	0.0497*	
H5B	0.78917	0.35099	0.33472	0.0497*	
H6	0.94896	0.67271	0.55625	0.0469*	
H7A	0.80965	0.93295	0.36434	0.0455*	
H7B	0.88352	0.76609	0.22023	0.0455*	
H8A	0.51074	0.79800	0.06399	0.0409*	
H8B	0.39057	0.73763	0.26622	0.0409*	

### Atomic displacement parameters (Å^2^)

**Table:**

	*U*^11^	*U*^22^	*U*^33^	*U*^12^	*U*^13^	*U*^23^
O1	0.0402 (9)	0.0388 (9)	0.0490 (10)	0.0100 (7)	0.0025 (7)	−0.0051 (7)
O2	0.0475 (11)	0.0688 (13)	0.1084 (19)	0.0249 (10)	−0.0090 (11)	−0.0466 (13)
O3	0.0573 (10)	0.0406 (9)	0.0293 (8)	0.0122 (7)	0.0094 (7)	0.0006 (7)
O4	0.0380 (8)	0.0414 (9)	0.0320 (8)	0.0167 (7)	0.0106 (6)	0.0001 (7)
C1	0.0380 (12)	0.0476 (12)	0.0379 (12)	0.0158 (10)	−0.0004 (9)	−0.0073 (10)
C2	0.0357 (11)	0.0408 (12)	0.0448 (13)	0.0158 (9)	0.0042 (10)	−0.0056 (10)
C3	0.0343 (10)	0.0324 (10)	0.0265 (10)	0.0126 (8)	0.0087 (8)	0.0002 (8)
C4	0.0492 (12)	0.0295 (10)	0.0346 (12)	0.0093 (9)	0.0103 (10)	0.0032 (9)
C5	0.0574 (14)	0.0424 (12)	0.0315 (12)	0.0253 (11)	0.0073 (10)	0.0079 (9)
C6	0.0381 (11)	0.0468 (13)	0.0322 (12)	0.0151 (10)	0.0025 (9)	0.0006 (9)
C7	0.0422 (12)	0.0336 (10)	0.0352 (12)	0.0069 (9)	0.0097 (9)	0.0024 (9)
C8	0.0412 (12)	0.0311 (10)	0.0322 (12)	0.0138 (9)	0.0076 (9)	0.0045 (9)

### Geometric parameters (Å, °)

**Table:**

O1—C1	1.313 (3)	C6—C7	1.524 (3)
O2—C1	1.205 (3)	C7—C8	1.520 (3)
O3—C6	1.441 (3)	C2—H2A	0.9700
O4—C3	1.443 (3)	C2—H2B	0.9700
O1—H1	0.83 (3)	C4—H4A	0.9700
O3—H3	0.86 (3)	C4—H4B	0.9700
O4—H4	0.83 (3)	C5—H5A	0.9700
C1—C2	1.507 (3)	C5—H5B	0.9700
C2—C3	1.523 (3)	C6—H6	0.9800
C3—C8	1.523 (3)	C7—H7A	0.9700
C3—C4	1.530 (3)	C7—H7B	0.9700
C4—C5	1.527 (4)	C8—H8A	0.9700
C5—C6	1.519 (3)	C8—H8B	0.9700
			
C1—O1—H1	112 (2)	H2A—C2—H2B	108.00
C6—O3—H3	111 (2)	C3—C4—H4A	109.00
C3—O4—H4	112 (2)	C3—C4—H4B	109.00
O1—C1—O2	122.7 (2)	C5—C4—H4A	109.00
O1—C1—C2	112.7 (2)	C5—C4—H4B	109.00
O2—C1—C2	124.7 (2)	H4A—C4—H4B	108.00
C1—C2—C3	114.80 (19)	C4—C5—H5A	109.00
O4—C3—C8	106.40 (17)	C4—C5—H5B	109.00
O4—C3—C4	109.61 (17)	C6—C5—H5A	109.00
C4—C3—C8	109.02 (15)	C6—C5—H5B	109.00
C2—C3—C4	112.02 (18)	H5A—C5—H5B	108.00
C2—C3—C8	110.55 (18)	O3—C6—H6	109.00
O4—C3—C2	109.09 (17)	C5—C6—H6	109.00
C3—C4—C5	111.86 (18)	C7—C6—H6	109.00
C4—C5—C6	112.5 (2)	C6—C7—H7A	109.00
O3—C6—C7	107.08 (19)	C6—C7—H7B	109.00
O3—C6—C5	110.6 (2)	C8—C7—H7A	109.00
C5—C6—C7	111.52 (18)	C8—C7—H7B	109.00
C6—C7—C8	112.19 (19)	H7A—C7—H7B	108.00
C3—C8—C7	112.17 (18)	C3—C8—H8A	109.00
C1—C2—H2A	109.00	C3—C8—H8B	109.00
C1—C2—H2B	109.00	C7—C8—H8A	109.00
C3—C2—H2A	109.00	C7—C8—H8B	109.00
C3—C2—H2B	109.00	H8A—C8—H8B	108.00
			
O1—C1—C2—C3	156.30 (19)	C2—C3—C8—C7	−179.47 (19)
O2—C1—C2—C3	−24.2 (3)	C4—C3—C8—C7	57.0 (2)
C1—C2—C3—O4	59.4 (2)	C3—C4—C5—C6	54.8 (3)
C1—C2—C3—C4	−62.1 (2)	C4—C5—C6—O3	67.6 (2)
C1—C2—C3—C8	176.07 (18)	C4—C5—C6—C7	−51.4 (3)
O4—C3—C4—C5	59.7 (2)	O3—C6—C7—C8	−69.5 (2)
C2—C3—C4—C5	−179.04 (19)	C5—C6—C7—C8	51.6 (3)
C8—C3—C4—C5	−56.4 (2)	C6—C7—C8—C3	−55.6 (2)
O4—C3—C8—C7	−61.2 (2)		

### Hydrogen-bond geometry (Å, °)

**Table:**

*D*—H···*A*	*D*—H	H···*A*	*D*···*A*	*D*—H···*A*
O1—H1···O3^i^	0.83 (3)	1.78 (3)	2.608 (3)	177 (3)
O3—H3···O4^ii^	0.86 (3)	1.90 (3)	2.756 (2)	175 (3)
O4—H4···O1^iii^	0.83 (3)	2.39 (3)	3.007 (3)	131 (3)
O4—H4···O2	0.83 (3)	2.20 (3)	2.789 (3)	128 (3)
C5—H5A···O1^iv^	0.97	2.60	3.511 (3)	157

Symmetry codes: (i) *x*−1, *y*−1, *z*−1; (ii) *x*, *y*, *z*+1; (iii) *x*+1, *y*, *z*; (iv) *x*+1, *y*, *z*+1.

## References

[bb1] Bernstein, J., Davis, R. E., Shimoni, L. & Chang, N.-L. (1995). *Angew. Chem. Int.* Ed. Engl. **34**, 1555–1573.

[bb2] Bruker (2005). *SADABS* Bruker AXS Inc., Madison, Wisconsin, USA.

[bb3] Bruker (2009). *APEX2* and *SAINT* Bruker AXS Inc., Madison, Wisconsin, USA.

[bb4] Farrugia, L. J. (1997). *J. Appl. Cryst.* **30**, 565.

[bb5] Farrugia, L. J. (1999). *J. Appl. Cryst.* **32**, 837–838.

[bb6] Jasinski, J. P., Butcher, R. J., Yathirajan, H. S., Mallesha, L., Mohana, K. N. & Narayana, B. (2009). *J. Chem. Crystallogr.* **39**, 777–780.

[bb7] Sheldrick, G. M. (2008). *Acta Cryst.* A**64**, 112–122. 10.1107/S0108767307043930 18156677

[bb8] Spek, A. L. (2009). *Acta Cryst.* D**65**, 148–155. 10.1107/S090744490804362X PMC2631630 19171970

[bb9] Vasudev, P. G., Rai, R., Shamala, N. & Balaram, P. (2008). *Biopolymers*, **90**, 138–150. 10.1002/bip.20957 18273891

